# Helical Klinotactic Locomotion of Two‐Link Nanoswimmers with Dual‐Function Drug‐Loaded Soft Polysaccharide Hinges

**DOI:** 10.1002/advs.202004458

**Published:** 2021-02-15

**Authors:** Jiaen Wu, Bumjin Jang, Yuval Harduf, Zvi Chapnik, Ömer Bartu Avci, Xiangzhong Chen, Josep Puigmartí‐Luis, Olgac Ergeneman, Bradley J. Nelson, Yizhar Or, Salvador Pané

**Affiliations:** ^1^ Multi‐Scale Robotics Lab Institute of Robotics and Intelligent Systems ETH Zurich Tannenstrasse 3 Zurich CH‐8092 Switzerland; ^2^ Faculty of Mechanical Engineering Technion – Israel Institute of Technology Haifa 32000 Israel; ^3^ Departament de Ciència dels Materials i Química Física Institut de Química Teòrica i Computacional Barcelona 08028 Spain; ^4^ ICREA Pg. Lluís Companys 23 Barcelona 08010 Spain

**Keywords:** drug nanoreservoirs, klinotactic locomotion, layer‐by‐layer, soft nanorobotics, template‐assisted electrodeposition

## Abstract

Inspired by the movement of bacteria and other microorganisms, researchers have developed artificial helical micro‐ and nanorobots that can perform corkscrew locomotion or helical path swimming under external energy actuation. In this paper, for the first time the locomotion of nonhelical multifunctional nanorobots that can swim in helical klinotactic trajectories, similarly to rod‐shaped bacteria, under rotating magnetic fields is investigated. These nanorobots consist of a rigid ferromagnetic nickel head connected to a rhodium tail by a flexible hydrogel‐based hollow hinge composed of chemically responsive chitosan and alginate multilayers. This design allows nanoswimmers switching between different dynamic behaviors—from in‐plane tumbling to helical klinotactic swimming—by varying the rotating magnetic field frequency and strength. It also adds a rich spectrum of swimming capabilities that can be adjusted by varying the type of applied magnetic fields and/or frequencies. A theoretical model is developed to analyze the propulsion mechanisms and predict the swimming behavior at distinct rotating magnetic frequencies. The model shows good agreement with the experimental results. Additionally, the biomedical capabilities of the nanoswimmers as drug delivery platforms are demonstrated. Unlike previous designs constitute metallic segments, the proposed nanoswimmers can encapsulate drugs into their hollow hinge and successfully release them to cells.

## Graphical abstract

A multifunctional nanorobot consisting of a magnetic head linked by a smart polymeric hinge to a nonmagnetic tail is investigated. The design allows this nanorobot to switch its dynamic behaviors from in‐plane tumbling to helical klinotactic swimming, depending on the frequency of rotating magnetic fields. A theoretical model for the nanorobot is developed. Its capability of drug delivery is demonstrated.

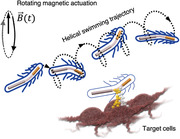

## 1 Introduction

Helical motion is a ubiquitous form of propulsion of motile microorganisms ranging from bacteria, protists, spores to invertebrate larvae, micrometazoa and several sperm cells of animals.^[^ ^1^ ^]^ Some microorganisms such as *Salmonella enteriditis*, *Helicobacter pylory*, or *Euglena gracilis* achieve corkscrew‐like motion by rotating their helical exoflagella. Other species such as *Spirochetes* twist their entire helical body by means of their endoflagella, resulting in corkscrew swimming. The nonreciprocal nature of the corkscrewing mechanism enables the translational motion of these microorganisms in low Reynolds number regimes as the time‐reversal symmetry is broken when rotating a chiral structure such as a flagellum.^[^ ^2^ ^]^ Interestingly, other microorganisms and cells such as ciliated bacteria, zooplankton, or sperm cells, while not necessarily exhibiting helical morphologies or organelles that can shape into helices, exhibit helical trajectories^[^ ^3^ ^]^ or ribbons.^[^ ^4^ ^]^ In this case, the time reversal symmetry can be broken by another mechanism (i.e., beating flexible organelles such as cilia, flagella, or other extremities) other than by rotating a chiral appendage. Recently, investigations comparing the swimming behavior of helical and rod‐shaped bacteria have shown that both types of bacteria swim in helical trajectories, and demonstrate that the helical structure only contributes at most 15% of the propulsion speed.^[^ ^5^ ^]^ Other studies show that the helical trajectory is a consequence of the periodic beating pattern of the propulsive appendages.^[^ ^6^ ^]^ This helical forward path, which is a consequence of a wavering side‐to‐side motion of the entire body or parts of it in response to an external stimulus, is known as helical klinotaxis, a term that was first introduced by Crenshaw and Edelstein‐Keshet.^[^ ^1^, ^7^ ^]^ While evolutionary biology has not yet shed light on the advantages of helical path swimming of several microorganisms, recent studies indicate that this type of motion pattern allows for an optimal control over directionality in contrast to straight path swimming,^[^ ^8^ ^]^ which is more sensitive to random variations of stimuli taking place intra‐ or extracellularly. Helical motion endows microorganisms with the ability to direct their positioning toward external stimuli without any biased random walk, and thus provides an efficient reorientation behavior to follow environmental cues.^[^ ^1^, ^9^ ^]^

Inspired by the motion of bacteria and other microorganisms, researchers have developed artificial micro‐ and nanodevices that can perform corkscrew motion and helical‐path swimming upon a suitable stimulation with external sources of energy. These small‐scale helical devices, also known as artificial bacterial flagella (ABFs)^[^ ^10^ ^]^ or helical micro‐ and nanorobots, are attracting a lot of interest because of their great potential for disease diagnosis,^[^ ^11^ ^]^ minimally invasive surgery,^[^ ^12^ ^]^ telemetry,^[^ ^13^ ^]^ targeted therapies,^[^ ^14^ ^]^ or plasmonic‐based nanorheology.^[^ ^15^ ^]^ The most common strategy consists of actuating magnetically responsive structures comprising helical components that revolve around their long axis when they are actuated by means of rotating magnetic fields resulting in a corkscrew locomotion.

**Figure 1:**
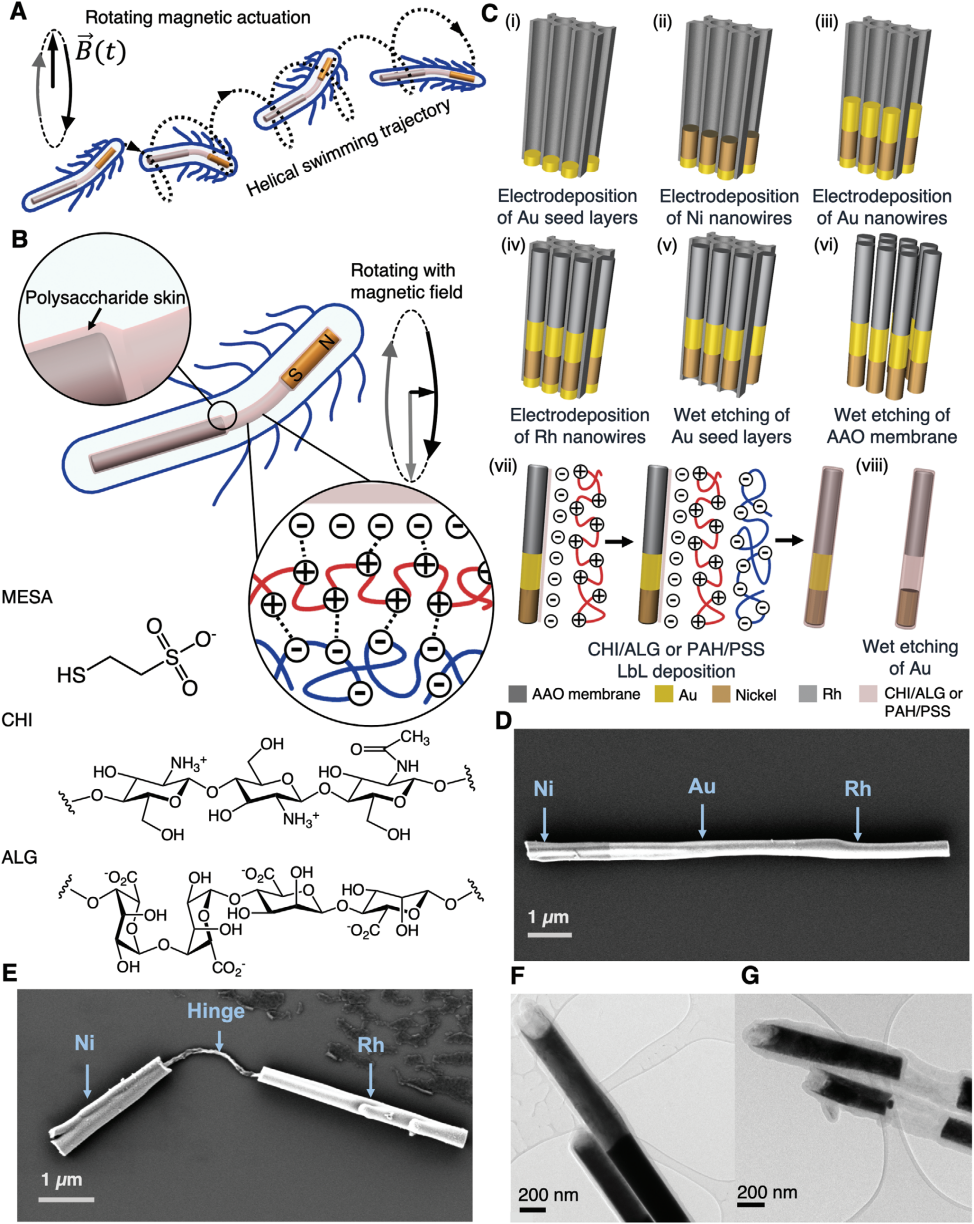
Schematic illustration of soft polysaccharide hinged nanoswimmer. A) Schematic drawings depicting the helical klinotactic swimming trajectory of soft‐hinged nanoswimmers under purely rotating magnetic actuation. B) Schematic illustration of rod‐shaped nanoarchitecture of an artificial nanoswimmer with polysaccharide skin and hinge. C) Fabrication process of flexible hinged nanoswimmers: i) electrodeposition of Au seed layers; ii) electrodeposition of Ni nanowires; iii) electrodeposition of Au nanowires; iv) electrodeposition of Rh nanowires; v) wet etching of Au seed layers; vi) wet etching of AAO membrane; vii) bilayers of CHI/ALG or PAH/PSS LbL deposition; viii) wet etching of Au. Characterization of hinged nanoswimmers: D) An SEM image showing three segments of Ni/Au/Rh after LbL self‐assembly of CHI/ALG bilayers. E) An SEM image of the finalized structure of nanoswimmer after Au etching. F) A TEM image showing a uniform bilayers of polysaccharide CHI and ALG before Au etching. G) A TEM image showing a uniform hollow hinge structure of finalized nanoswimmers.

The incorporation of a complex helical body shape in swimmers’ architectures is not necessary to enable helical swimming. This is an advantageous feature of the presented hinged swimmers, especially in terms of fabrication. While there is a wealth of approaches for fabricating small‐scale helices, control over features such as pitch and turns of the helical body shapes can become very challenging at micro‐ and nanoscale. Note that these geometric parameters can severely affect not only the swimmers’ locomotion but also their interaction with cells.^[^ ^16^ ^]^ In this vein, adjusting the length of hinges and rods for nonhelical‐shaped swimmers becomes easier than helices from the manufacturing point of view. In this contribution, we investigated for the first time the locomotion of a highly integrated multifunctional nonhelical nanorobot made of segmented nanowires. It is capable of helical klinotactic swimming when stimulated by purely rotating magnetic fields (**Figure** **1**A). The nanoswimmer consists of two cylindrical rigid metallic links (nickel and rhodium) joint by a soft polymeric hinge (Figure 1B). The ferromagnetic nickel link serves as the magnetically responsive motile head component. The nonferromagnetic rhodium segment acts as a tail, while the polymeric hinge functions as a flexible joint to promote the motion of the swimmer by deformation. Compared to helical nanoswimmers, hinged rod‐shaped swimmers display a richer spectra of motion mechanisms. Apart from the helical path swimming hereby demonstrated, this type of small‐scale robots can swim by beating their tails when actuating them under oscillating magnetic fields,^[^ ^17^ ^]^ mimicking the motion of eukaryotic cells such as spermatozoa. Those mode‐changing capabilities not only can trigger different functionalities such as optimal translation or on‐demand therapeutic cargo release,^[^ ^18^ ^]^ but also provide swimmers with higher adaptability in changing swimming environments such as the human body. A theoretical model is afterward developed to analyze the propulsion mechanism, and predict the helical swimming behavior as function of the rotating magnetic frequency, showing a good agreement between the simulations and the experimental results. The helical klinotactic path of the nanorobots is a consequence of the periodic conical rotations of the head and the tail.

Significant efforts are being directed in incorporating soft structures in micro‐ and nanoswimmers that not only can enable sophisticated locomotion mechanisms^[^ ^17^ ^]^ but also can perform other tasks such as cargo loading, degradation or stimuli‐triggered drug release. Here, we propose the use of chemically responsive polysaccharide hinges made of alternate layers of chitosan and alginate (Figure 1B). Compared to previous designs made of fully metallic assemblies,^[^ ^19^ ^]^ our motile nanosystems are able to encapsulate therapeutic cargoes in the hollow cavity of the hinges, thus not limiting the loading of the therapeutic agent to the surface. We also performed preliminary in vitro cell experiments to demonstrate that the loaded cargo in the polysaccharide hinge can be released to target cells.

## 2 Results and Discussion

### 2.1 Fabrication and Characterization

The hinged nanoswimmers were synthesized by a combination of template‐assisted electrodeposition^[^ ^17^ ^]^ and layer‐by‐layer (LbL) self‐assembly deposition.^[^ ^20^ ^]^ The manufacturing sequence is schematically depicted in Figure 1C. First, a sacrificial gold (Au) seed layer was evaporated on one side of a commercially available anodized aluminum oxide (AAO, thickness ≈ 60 µm; pore diameter ≈ 200 nm) membrane to create an electrical contact for subsequent plating operations. Next, a 3 µm thick Au plug layer was grown on the Au seed layer by electrodeposition in the pores of the AAO membrane. The heads and tails of the nanoswimmers were synthesized by plating a multilayer of ferromagnetic nickel (Ni), Au, and nonferromagnetic rhodium (Rh). Note that Rh and Ni were chosen considering their facile platabilities, the efficiency of their electrodeposition within confined nanocavities, and their inertness toward the final etching procedure for the formation of the hinged structures. Additionally, Rh is weakly paramagnetic and thus is only responsive to strong external magnetic fields. In this way, we ensure that only Ni will react during the manipulation of the structures by means of external magnetic fields. The Au plug layer and the AAO membrane template were then selectively etched, releasing multisegmented Au–Ni–Rh nanowires. Subsequently, a monolayer of mercaptoethanesulfonic acid (MESA) was functionalized on the nanowire surface through metal‐thiolate bonds to render the surface of the multimetallic nanowires with a negative charge. Two types of LbL coatings were used in this work: chitosan (CHI): alginate (ALG), and polyallylamine hydrochloride (PAH):polystyrene sulfonic acid (PSS). On the negatively charged surface of the nanowires, thin layers of positively charged polysaccharide CHI (or PAH) and negatively charged ALG (or PSS), which are biodegradable, were alternately deposited. As LbL is based on growing oppositely charged polymer chains, the surface potential of the nanowire was monitored after every consecutive polyelectrolyte treatment to ensure the success of each LbL step. Figure S1A in the Supporting Information shows the change of *ζ*‐potential for CHI and ALG (Figure S1B, Supporting Information: PAH and PSS) after deposition of each layer up to eight bilayers. It shows that the measured *ζ*‐potential was negative after the first monolayer MESA deposition. Afterward, the surface polarity changed alternately when each new layer of polyelectrolyte was deposited. Sign changes of *ζ*‐potential after each layer deposition is an indication of a successful stepwise and alternating CHI and ALG (PAH and PSS) layer deposition. After LbL self‐assembly deposition, the Au layer in the middle of the nanowires was selectively etched away to create a hollow nanostructure, which can function as both a flexible hinge and a drug reservoir. The final desired hinged nanoarchitecture was achieved, and this was referred to as an artificial flexible hinged nanoswimmer.

The morphology of these nanoswimmers was characterized by scanning electron microscope (SEM) and transmission electron microscope (TEM). Figure 1D presents the secondary electron SEM images of nanowires after LbL self‐assembly deposition of CHI/ALG on the segmented Ni–Au–Rh nanowires. In Figure 1E, the final structure of a flexible hinged nanoswimmer after wet etching of the Au segment in Figure 1D can be clearly observed. It consists a 2.26 µm long Ni nanohead connected through a 2‐µm long flexible polysaccharide hinge composed of eight bilayers of CHI/ALG to a 3.6‐µm long Rh nanotail. The collapsed aspect of the hinge in the SEM picture (Figure 1E) is caused by the dehydration of the hydrogels composing the hinge during the sample preparation. Figure 1F,G presents the TEM image of nanowires obtained before Au etching and after Au etching, respectively. The total thickness of the uniform coating of CHI/ALG bilayers is ≈64 nm. Note that this fabrication technique allows for fabricating hinged nanoswimmers with different configurations and materials.

### 2.2 Locomotion Performance

**Figure 2:**
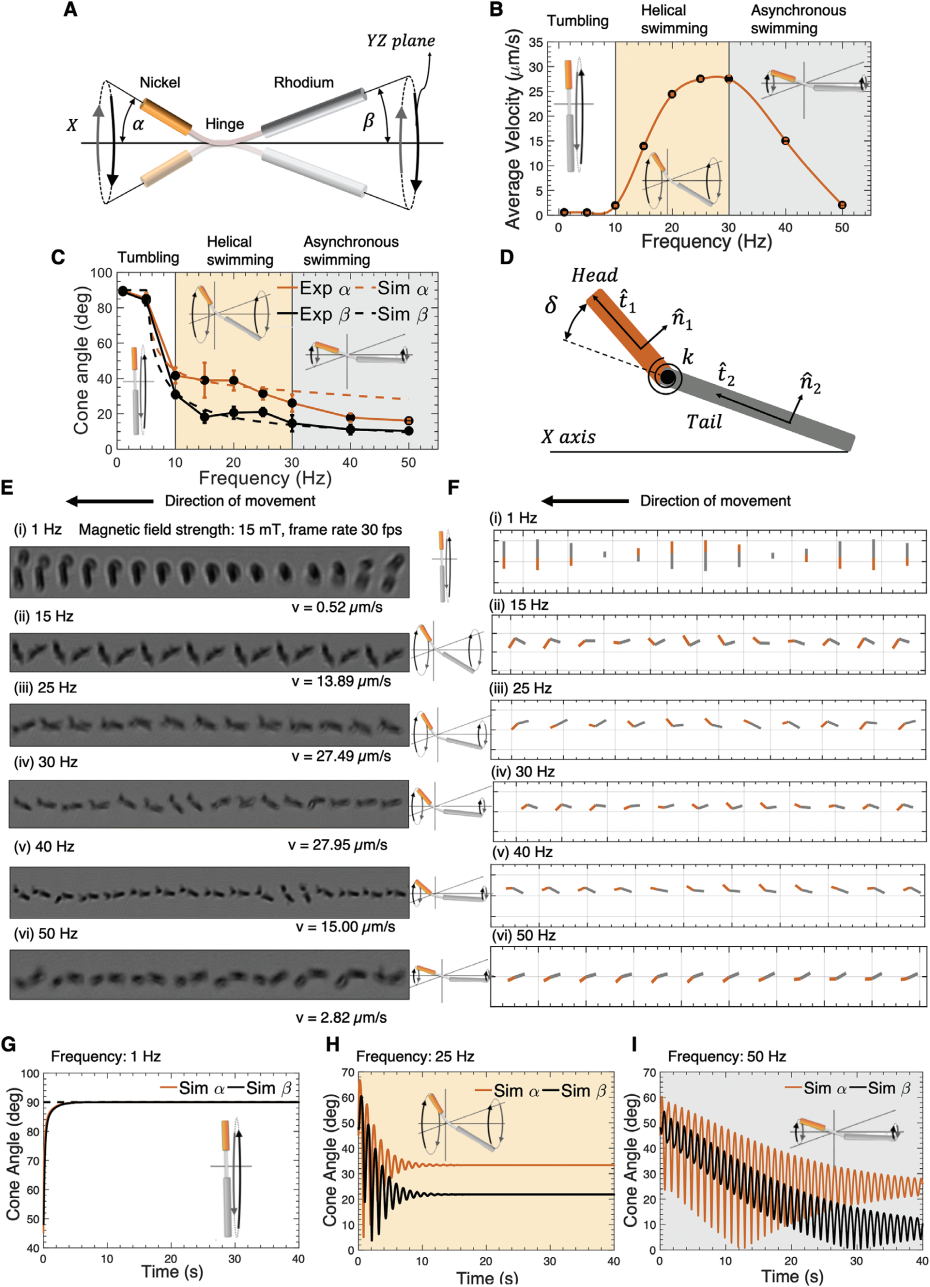
A) A schematic diagram of nanoswimmer illustrating precession angles and XYZ coordinate. B) Experimental values of average speed of a flexible hinged nanoswimmer as a function of rotating magnetic frequency at field strength of 15 mT. C) Mean precession cone angles *α* and *β* as a function of the external rotating magnetic field frequency in the experiment. D) A schematic illustration depicts the simplified model of nanowire. E) Dynamic motion sequences of the nanoswimmer at different frequencies (1, 15, 25, 30, 40, and 50 Hz) under a constant magnetic field of 15 mT captured at 30 fps, which means the time interval between two motion is 33 ms. The swimming direction was from right to left. F) Numerical simulation results for the effect of magnetic field frequencies on nanoswimmer's speed under rotating magnetic field. Image sequences of motion dynamics of the nanoswimmers at distinct frequencies (1, 15, 25, 30, 40, and 50 Hz) at a constant magnetic field strength of 15 mT. G–I) Precession cone angles *α*(*t*) and *β*(*t*) as a function of locomotion time under three regions: G) wobbling region with frequency 5 Hz; H) helical swimming region with frequency 25 Hz; I) asynchronous region with frequency 50 Hz. All bar plots represent average ± standard deviation (SD) (*n* = 5).

To study the locomotion behavior of nanoswimmers, we define the following parameters as shown in the schematic diagram in **Figure** **2**A. *YZ* plane corresponds to the plane of the magnetic field rotation. *α* is the maximum angle between the magnetic head and the *X* axis, and *β* is the maximum angle between the tail link and the *X* axis. The locomotion of nanoswimmers was characterized in a 65% glycerol solution under a Magnebotix Nanomag setup, coupled to an inverted optical microscope.^[^ ^21^ ^]^ The setup consists of eight coils in a hemispherical order, which can generate magnetic fields of up to 50 mT and magnetic field frequencies of up to 100 Hz. For the analysis of the locomotion characteristics of the nanoswimmers, magnetic fields of 15 mT and rotational frequencies of up to 50 Hz with steps of 5 Hz were investigated first. Figure 2B shows the average swimming speed of two‐link nanoswimmers with CHI/ALG hinges as a function of different rotating magnetic frequencies. The lengths of the segments comprising the nanoswimmers were ≈2, ≈2, and 3.5 µm for the Ni head, the flexible hinge, and the Rh tail, respectively, resulting in a total length of 7.5 µm. From Figure 2B, a resonant‐like speed profile can be observed. At low frequency below 10 Hz, the movement speed of the nanoswimmer was quite low at around 0.5 µm s^−1^. The nanoswimmer was bent a little and rotated in YZ plane. From 10 to 30 Hz, it started to move forward in a helical path, as shown in Video S1 in Supporting Information. The speed increased almost linearly up to 27.49 µm s^−1^ in the frequency region of 10–25 Hz. At the frequency range of 25–30 Hz, the speed curve shows an inflection point corresponding to a maximum speed of around 28 µm s^−1^. A further increase in the frequency results in a decrease of the speed toward zero. Similar results were observed for PAH:PSS hinged nanoswimmers (Figure S2, Supporting Information).

To better understand the locomotion of the swimmers, the measured precession angles *α* and *β* were plotted in Figure 2C. We classify the precession behavior into three different motion phases: tumbling, helical and asynchronous swimming, based on the speed and precession angles of the swimmer, as shown in Figure 2B,C. The transformation of the locomotion of the nanoswimmer from tumbling to helical motion is supported by the fact that the precession angle *α* and *β* dramatically decrease as the rotational magnetic frequency increases. In Figure 2E, the representative movement sequences are captured for the three motion phases under the magnetic field strength of 15 mT at different rotating frequencies of 1, 15, 25, 30, 40, and 50 Hz. The nanoswimmer's movement videos under these frequencies can be found in Videos S2–S7 (Supporting Information). At low frequencies, the Ni head of the nanoswimmer aligns its easy magnetization axis (long axis of the Ni segment) parallel to the direction of the external magnetic field. When the magnetic field rotates, the nickel head tumbles around its short axis synchronously. As the frequencies are low, the rotation of the hinge and the tail of the nanoswimmer occur in the same plane of the rotating field. In this case, the swimmer is slightly flexed at the hinge, and keeps the bending angle (i.e., the angle between the magnetic head and the tail appendage) constant while tumbling (Figure 2E‐i). In this state, the nanoswimmer presents almost no net propulsion. When the rotational frequency of the applied magnetic field increases to 15 Hz (Figure 2E‐ii), the Ni head is no longer able to align parallel with the field in the *YZ* plane. Instead, the head rotates conically around the *X* axis with a precession angle *α*. Because the Ni head is connected through the polymeric hinge to the tail, the torque experienced by the Ni segment is transferred to the flexible polymer hinge, and resulting in the conical rotation of the Rh tail along the horizontal axis (*X*) with a precession angle *β*. The difference between the precession angles *α* and *β* caused by the hinge flexibility breaks the front‐back symmetry. This front and back asymmetry creates an “effective chirality” equivalent to screw, which generates propulsion along the axis of rotation *X*.^[^ ^22^ ^]^ When the frequency rises to 25 Hz (Figure 2E‐iii), the purely spatial helical motion can be observed leading to a high speed of the nanoswimmer. If the frequency is further increased, the swimmer can no longer rotate in‐sync with the rotating magnetic field and the transition to the asynchronous regime take place. The step‐out frequency, where the maximum magnetic torque is reached, appears at around ≈30 Hz (Figure 2E‐iv). Figure 2E‐v shows a sequence of motion of a two‐link nanoswimmer at the asynchronous regime using a frequency of 40 Hz. During forward locomotion, the pure spatial helical motion is disturbed by a more complicated nonlinear movement, which can be described as an uncontrollable swirling. In this region, the propulsion speed drops dramatically. As the frequency increases above the step‐out frequency, the resistive drag torque exceeds the maximum magnetic torque, causing the nanoswimmer to swim unstably until the forward propulsion speed further diminishes to zero. Very small propulsion can be observed at a frequency of 50 Hz (Figure 2E‐vi). In this region, the swimming behavior is unstable as the nanoswimmer is not controllable for directed propulsion.

To systematically understand the theoretical mechanism of the spatial dynamic motion of the swimmers, an analytical model was developed. The theoretical model of the two‐link nanoswimmer comprises two rigid slender cylinders connected by a point‐size revolute joint, with a relative angle *δ* of rotation about a body‐fixed axis perpendicular to the link's longitudinal direction (Figure 2D). To simplify the calculations, the hinge is considered as a “point hinge” with zero length. Flexibility of the revolute joint is represented by a torsion spring with a linear stiffness *k*, such that the torque at the joint is given by *τ*_*k*_ =   − *kδ*. The bending stiffness *k* of the hinge is estimated as $k=\frac{EI}{l_{h}}$, where *E* is the Young's modulus of the polymer hinge, *I* is the second moment of area for hinge, and *l*_h_ is the length of the real hinge. Since the hinge is a round hollow structure, *I* can be calculated as $I=\frac{\pi }{64}\left[{\left(d_{o}\right)}^{4}-{\left(d_{i}\right)}^{4}\right]$, where *d*_o_ is outer diameter of the hinge, and *d*_i_ is inner diameter. The head link is made of ferromagnetic material and its magnetization vector **m** is directed along the link's longitudinal axis ${\hat{t}}_{1}$, while being actuated by a rotating magnetic field. The external magnetic field ***B*** (*t*) is rotating in *Y*–*Z* plane and is given by $B\left(t\right)=\left[\begin{array}{ccc} 0 & \sin(\omega t) & \cos(\omega t) \end{array}\right]\;\cdot B$, where *B* is the magnetic field's intensity and *ω* is its angular velocity, or rotational frequency. Assuming a spatially uniform magnetic field, it induces a pure torque ***τ*** ^m^ on the magnetized head link, which depends on its orientation and the time‐varying field **B** (*t*) as ***τ*** ^m^ =  **m** × **B**. The nanoswimmer is submerged in a viscous fluid. The Stokes drag forces and torques acting on each of the two links **f**_*i*_,***τ***_*i*_ depend linearly on their angular velocity vectors **v**_*i*_,**Ω**_*i*_. Neglecting hydrodynamic interactions between the links implies linear drag resistance relations, which are represented by **f**_*i*_ =   − **R**_*i*_ **v**_*i*_ and ***τ***_*i*_ =   − **Q**_*i*_ **Ω**_*i*_. When the velocity vectors **v**_*i*_,**Ω**_*i*_ of each link are expressed in body‐fixed reference frames aligned with the link's longitudinal axes ${\hat{t}}_{i}$, the resistance matrices **R**_*i*_,**Q**_*i*_ are constant and diagonal. We used known formulation of resistance matrices for slender prolate spheroids,^[^ ^23^ ^]^ see the Supporting Information for further details. The vector of generalized coordinates describing the swimmer's spatial pose is chosen as **q** =(*x*, *y*, *z*, *φ*, *θ*, *ψ*, *δ*)^*T*^ , where (*x*, *y*, *z*) denote the position of the head link's center, (*φ*, *θ*, *ψ*) are Euler angles describing its spatial orientation, and *δ* is the joint angle, see the Supporting Information for details. This enables formulating the links’ linear and angular velocities **v**_*i*_,**Ω**_*i*_ in terms of orientation and generalized velocities, **q** and $\dot{q}$. Static equilibrium balance of forces and torques on the two links, including viscous drag terms **f**_*i*_,***τ***_*i*_, magnetic torque ***τ*** ^*m*^ and elastic joint torque ***τ***_*k*_, leads to a system of first‐order nonlinearly coupled ordinary differential equations of the form $\dot{q}=\;g\left(q,t\right)$, see the Supporting Information for detailed derivation. This system is integrated numerically using **ode45** function in MATLAB, under initial conditions **q**(0) =  0 of straightened links aligned with X axis. The swimmer's motion is extracted from solutions of **q**(*t*) after convergence to steady‐state synchronized periodic motion, whenever it exists. Beyond the step‐out frequency, such synchronized motion no longer exists, and we observe quasi‐periodic oscillations.

Figure 2F shows representative numerical simulation results of the nanoswimmer's moving sequences projected to *X*–*Y* plane under distinct frequencies: 1, 15, 25, 30, 40, and 50 Hz. The orange link represents the ferromagnetic Ni head, and gray link represents the Rh appendage. The precession cone angles are defined as $\alpha \;=\cos^{-1}\left({\hat{t}}_{1}\cdot \hat{x}\right)\;$ and $\beta \;=\cos^{-1}\left({\hat{t}}_{2}\cdot \hat{x}\right)$. In Figure 2G–I, time plots of these cone angle *α*(*t*) and *β*(*t*) are shown for the same simulation under three regions: tumbling (1 Hz), helical (25 Hz), and asynchronous (50 Hz) swimming. Those simulated swimmer's movement video under three frequencies can be found in Videos S8–S10 (Supporting Information). We could notice that at a tumbling frequency of 1 Hz (Figure 2F‐i) the nanoswimmer aligns with the direction of the rotating magnetic field undergoing planar rotation (tumbling) around *X* axis in *YZ* plane. *α*(*t*) and *β*(*t*) angles converged to 90° quickly for *YZ* plane tumbling (Figure 2G). No obvious translational velocity at *X* direction can be observed. In Figure 2F‐iii, the helical motion under 25 Hz was plotted. The nanoswimmer cannot align with the magnetic fields. The precession angles *α*(*t*) and *β*(*t*) converge to constant nonzero values after initial oscillation, as shown in Figure 2H. There is always an angle difference between the Ni head (orange) and the Rh tail (gray). When the locomotion of the swimmer reaches steady state, the head's precession angle *α* is constantly larger than the tail's angle *β*, as expected. If the frequency is increased even further to the asynchronous region (as shown in Figure 2F‐vi with a frequency of 50 Hz), the nanoswimmer swings with a small amplitude on the horizontal axis. *α* and *β* shown in Figure 2I continuously oscillate around 20° and 10°, because of the nonsync motion at the region beyond the step‐out frequency. The simulated oscillating values of precession angles *α* and *β* agree with those observed experimentally (16.10° and 10.39°) as shown in Figure 2C.

**Figure 3:**
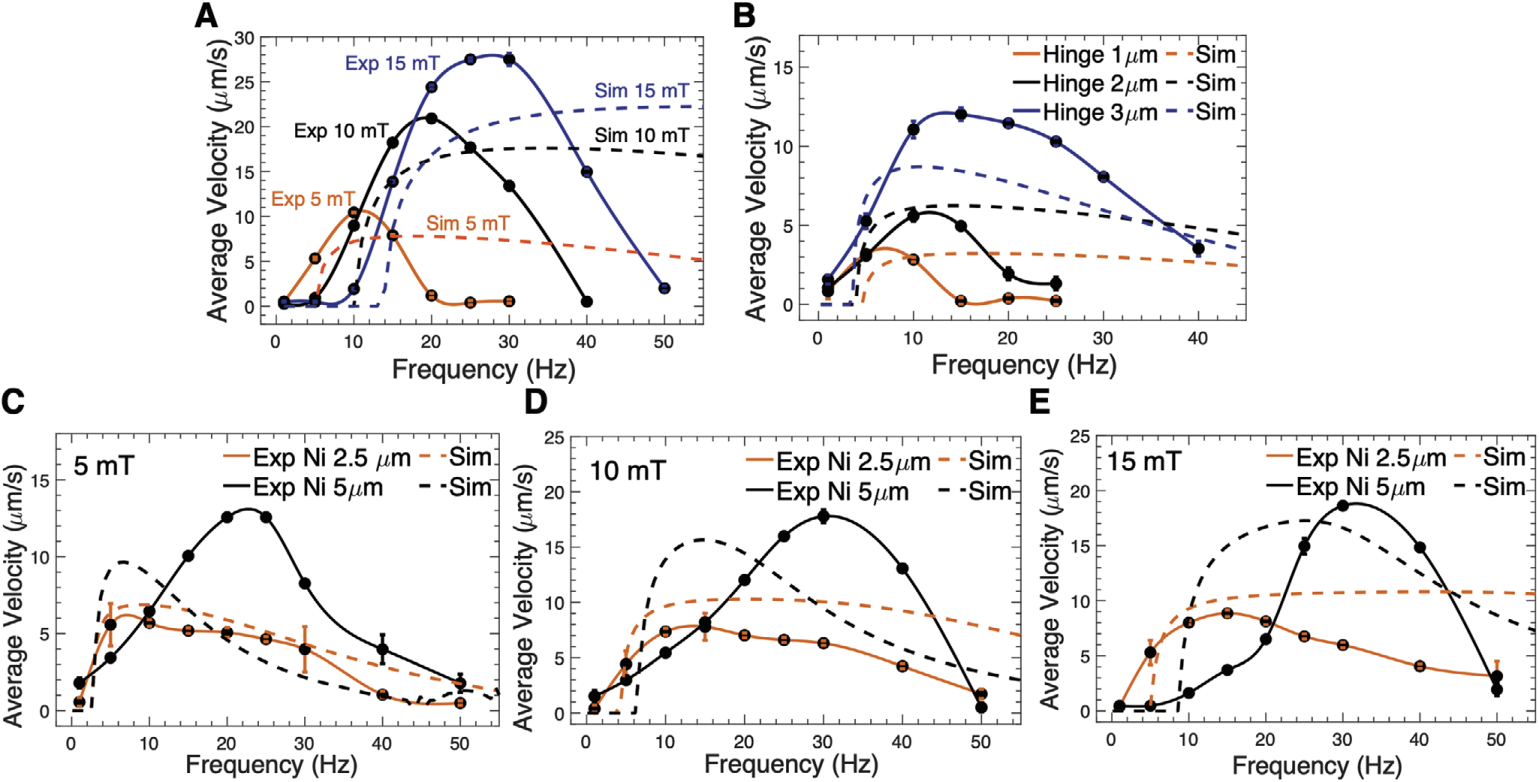
A) The average speed of hinged nanoswimmers with the Ni head ≈2 µm, the CHI/ALG hinge ≈2 µm, and the Rh tail ≈3.5µm as a function of rotating magnetic frequency at field strength 5, 10, and 15 mT. The dimensions for the Ni head and the Rh tail were chosen to be 3 and 4.5 µm in the simulation. B) The effect of hinge length on the speed of the nanoswimmers under a rotating magnetic field of 5 mT. Nanoswimmers with the same length of the Ni head ≈2.5 µm, the Rh tail ≈4 µm, and the CHI/ALG hinge with varying lengths of 1.0, 2.0, and 3.5 µm were used for the experiment. In the simulation, the dimensions of the Ni head were chosen to be 3, 3.5, 4.3 µm and the Rh tail were chosen to be 4.5, 5, 5.8 µm, respectively. C–E) The effect of the Ni length on the average speed of the nanoswimmers, actuated at D) 5 mT, E) 10 mT, and F) 15 mT. Black solid lines: Ni head (≈2.5 µm)/CHI/ALG hinge (≈3.5 µm)/Rh tail (5 µm). Orange solid lines: Ni head (≈5 µm)/CHI/ALG hinge (≈3.5 µm)/Rh tail (≈5 µm). Black dotted lines: Ni head (4.4 µm)/Rh tail (6.7 µm). Orange dotted lines: Ni head (8 µm)/Rh tail (6.7 µm). Solid line: experiment results, dotted line: simulation results. All bar plots represent average ± SD (*n* = 5).

**Figure** **3**A shows the comparison between numerical simulations and experimental measurements of average swimming speeds as a function of frequency under different magnetic field strengths 5, 10, and 15 mT. In the experiments, the lengths for the Ni head, the flexible hinge, and the Rh tail of the nanoswimmers were ≈2, ≈2, and 3.5 µm, respectively, resulting in a total length of 7.5 µm. As the theoretical model assumes a “point hinge” with zero length, the real hinge length was split into half and added each to the rigid Ni head and Rh tail in the simulation in order to match the same total length of the nanoswimmer. The dimensions for the Ni head and the Rh tail were then chosen to be 3 and 4.5 µm in the simulation. From the magnetic manipulation experiments, one can see that the speed of the hinged nanoswimmer not only depends on the frequency of the rotating magnetic field, but also on the strength *B* of the applied magnetic field. A resonant‐like speed profile can be observed at all three magnetic field strengths of 5, 10, and 15 mT. A higher strength of the magnetic field shifts the resonant‐like speed profile to a higher frequency range and increases the maximum speed. The in‐plane rotation can be observed at 1z for 5 mT, 1–5 Hz for 10 mT, and 1–10 Hz for 15 mT. The step‐out frequency at different field strengths and the corresponding maximum velocities observed are as follows: 10.44 µm s^−1^ for 5 mT at 10 Hz, 20.90 µm s^−1^ for 10 mT at 20 Hz, and 27.95 µm s^−1^ for 15 mT at 30 Hz. The theoretical model agrees with the experiment in the low frequency range. While our simulations cannot predict quantitatively the values of maximum speed and the behavior of the swimmers above the step‐out frequency, the model captures the transition frequency from in‐plane tumbling to helical swimming and the step‐out frequency for all three magnetic field strength values. Additionally, the simulated speed also increases with increasing the magnetic field strength, which agrees with experimental results.

Later, we investigated the propulsion performance with varying the length of different parts. Figure 3B shows the effect of hinge length on the speed of the nanoswimmer as a function of the frequency by varying the hinge length (1.0, 2.0, 3.5 µm) at a rotating magnetic field of 5 mT. The lengths of the Ni head and the Rh tail were 2.5 and ≈4 µm. One can observe that, for longer swimmer hinges, the resonance frequency increases due to a decrease in the bending stiffness (or increase in the flexibility) of the hinge, leading to an increased swimming performance. In a system with a longer hinge, the coupling between the motion of the Ni head and that of the Rh appendage decreases. As the Ni head can move more independently from the Rh appendage, the magnetic component can rotate synchronously with the magnetic field even at higher frequencies. In this case, simulations show a good agreement for experiments with swimmers with shorter hinges, which seems reasonable because of the simplified assumption of point hinge. The model also captures the resonant‐like speed profiles and the in‐plane synchronization of the swimmer with the rotating magnetic field at low frequencies. Yet, the model cannot resolve the values of the step‐out frequency.

The effect of the Ni length on the speed was also investigated by varying the length of the Ni head (≈2.5 and ≈5 µm). Figure 3C–E shows the locomotion speed plot as a function of frequency under different magnetic fields (5, 10, and 15 mT). The lengths of the hinge and the tail were kept at ≈3.5 and ≈5 µm, respectively. One can see that swimmers with Ni heads of 5 µm exhibit maximum speeds of almost twice the values of those of swimmers with heads of 2.5 µm. These results agree with the fact that a double length of the magnetic Ni head leads to a double magnetic torque exerted on the swimmer. Additionally, we observe that the step‐out frequency of swimmers with the shorter Ni head is almost half of that of swimmers with double‐length heads. In our simulations, as we assume a point hinge, in order to maintain the same total length of the real nanoswimmers, we split the hinge's length *l*_h_ into half and added 0.5*l*_h_ to the length of both the head and the tail. Again, our simulation results capture the experimental resonant‐like speed profiles for both types of swimmers under three magnetic fields 5, 10, and 15 mT. For the shorter Ni head, the swimming performance from simulations and experiments are in good agreement. The model reproduces well the magnitude of the maximum speed, the transition frequency from in‐plane tumbling to corkscrew propulsion, and the step‐out frequency. In contrast, the simulations for swimmers with a longer Ni head, reveal that the simplifying assumption of “point hinge” no longer holds. More sophisticated models need to be investigated further.

### 2.3 Drug Loading and In Vitro Release Experiments in Cell Culture

**Figure 4:**
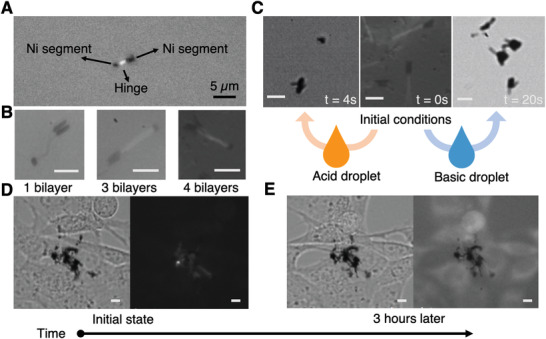
A) A fluorescence image of a nanoswimmer with 4 bilayers of CHI/ALG hinge after RhB loading. B) Fluorescence microscope images for different bilayers of CHI/ALG hinge. C) Demonstration of pH‐responsive drug release with CHI/ALG hinge under fluorescence microscope. D) Optical and fluorescent images of in vitro drug release experiment. The encapsulate of RhB can be observed as a glow inside the hollow hinge at initial state. E) After 3 h, fluorescent RhB diffused out of the hinge. Scale bars:  µm.

The hollow hinges of our nanodevices not only can serve for locomotion purposes, but they can also act as cargo reservoirs. As a proof of concept, here we show the potential of these swimmers as drug delivery nanoagents. To simplify the experiments and minimize possible changes of contrast between the two metallic segments under fluorescence microscope, we used nanoswimmers consisting of a Ni head, a polymeric hinge and a short Ni tail. A model fluorescent drug (rhodamine B (RhB)) was loaded in the polymeric hinges of swimmers with CHI/ALG bilayers. For this experiment, the fabricated swimmers were immersed in a 1 mg mL^−1^ solution of RhB and shaken for 12 h. Afterward the nanowires were thoroughly cleaned in DI water to remove the excess of RhB on the surface of the swimmers. The fluorescent dye can be observed as a glow in between the Ni and Rh and is observable as a dim glow in both polymer combination PAH/PSS and CHI/ALG as shown in **Figure** **4**A. Note that the dye appears to concentrate within the hollow hinge as brightness can only be detected in the hinge region rather than all around the Ni head or the Rh tail. We also investigated the influence of the number of bilayers on the fluorescent/loading of RhB. Interestingly, for a small number of bilayers, the dye cannot be detected in the fluorescence image at the hinge region (Figure 4B). In contrast, three‐bilayers is the minimum number for which RhB, can be observed. Probably, one or two bilayers are not enough to keep sufficient amount of RhB within the hinge during the rinsing procedure for removing the excess of dye. Next, we evaluated qualitatively the release behavior of the RhB‐loaded nanoswimmers in solutions with different pH. The nanoswimmers were first immersed in a solution with a pH around 5–6.6. Then, a droplet of citric acid solution (pH ≈ 2.5) was dispensed on the nanowires to lower down the pH. CHI/ALG nanoswimmers showed a quick release of RhB as evidenced from the captured fluorescent microscope images (Figure 4C). Considering the p*K*_a_ values of chitosan and alginate (p*K*_a,CHI _≈ 6.3, p*K*_a,ALG_ ≈ 3.4), at pH < 3.4, chitosan is in its cationic form with its amino groups protonated (—NH_3_^+^) while alginate is in its neutral form with its carboxylate groups protonated (—COOH). In these conditions, the electrostatic repulsion between chitosan and alginate layers leads to a significant swelling, ultimately causing RhB to diffuse out from the hinged reservoir. Additionally, we also observed the disintegration of the hinge, which is in agreement with previous studies.^[^ ^24^ ^]^ When adding a droplet of a basic solution of NaOH (pH > 10), the release of RhB was also observed but, in this case, was slightly slower as shown in the Figure 4C. In these conditions, amino groups in chitosan are in their neutral form (—NH_2_), while the carboxylates are in their anionic form (—COO^−^). Again, electrostatic repulsion could explain the diffusion of RhB out from the hinge. The slower release could be attributed to a lower degree of swelling and shrinkage of the layers in basic conditions.^[^ ^24^ ^]^ We performed similar experiments with nanoswimmers containing PAH/PSS hinges (Figure S3, Supporting Information). When exposing the nanoswimmers to an acidic droplet, release of RhB was observed after 4 s. The p*K*_a_ of PAH and PSS are p*K*_a,PAH _≈ 8.8 and p*K*_a,PSS_ ≈ 1.0, respectively. At low pH, PAH is in its cationic form with its protonated amino groups (—NH_3_^+^), while PSS is in its anionic form with its deprotonated sulfonate groups (—SO_3_^−^). The osmotic pressure increases in these conditions, water molecules diffuse into the polyelectrolyte, and thus the multilayers exhibit a high degree of swelling, which could explain the observed rapid release of RhB.^[^ ^25^ ^]^ At basic pH, on the contrary, the decrease of osmotic pressure leads to a shrinkage of the polyelectrolyte multilayered hinge.^[^ ^25^ ^]^ This deswelling effect makes the drug molecules trapped inside the hollow hinge, thus no significant RhB release was observed. A preliminary in vitro experiment was further investigated to confirm the drug release capability of the klinotactic nanoswimmers in a cell culture of mouse 3T3 fibroblast cells. Figure 4D,E presents the optical and fluorescent images of CHI/ALG hinged nanoswimmers with fibroblasts at the beginning and after three hours of incubation. It can be clearly observed that after 3 h, the fluorescent RhB encapsulated within the hinge diffused out and stained the cell. Therefore, CHI/ALG hinged nanoswimmers have the ability to deliver and release drugs to target cells, and could potentially be used as a drug carrier for targeted drug delivery.

## 3 Conclusions

In this study, a flexible hinged nanoswimmer with magnetic Ni head, soft hinge and Rh tail was successfully fabricated. First, a Ni–Au–Rh nanowire was fabricated by templated‐assisted electroforming, and then a polysaccharide multilayer of CHI/ALG (or polyelectrolyte PAH/PSS) was deposited through LbL self‐assembly on the nanowire. Selective Au removal formed a hollow nanohinge linking the magnetic head with the nanomagnetic tail. These nanorobots are capable of helical klinotactic swimming under pure rotating magnetic fields. The dynamic behavior of in‐plane tumbling and spatial helical klinotactic swimming can be switched by changing the magnetic field frequency and strength. The effects of the magnetic field strength, the Ni head length, and the hinge length on locomotion behavior were investigated in the experiments. In addition, a theoretical model was built to analyze the propulsion performance and improve the understanding of the swimming mechanisms. The simulation results were in a good agreement with the experimental results. Although this is not the first work of artificial robots that can exhibit multimode locomotion, this is the first work that has studied and revealed the nature of the transition of different locomotion modes by only changing the magnetic field without any additional stimulus. The successful loading of a fluorescent RhB in the soft hollow hinge of the swimmer and its successful release to a mouse fibroblast 3T3 target cell shows that the nanoswimmer has the capabilities of cargo transport and drug delivery.

## 4 Experimental Section

##### Electrodeposition of Segmented Nanowires

A commercially available AAO membrane (Anodisk 25, Whatman) with a thickness of 60 µm and a nominal pore diameter of 200 nm was used as template. A 200 nm layer of Au was first electron beam evaporated on one side of the AAO membrane in order to create an electrical contact. Then, a sacrificial Au plug layer with a thickness 3 µm was electroplated to prevent plating functional parts at the bottom part of the pores as they exhibit a highly branched terminal morphology. The membrane was stuck with a copper tape fixed onto a glass holder that hindered the solution from leaking behind the membrane. The glass holder was then submerged in a beaker filled with a cyanide‐based Au electrolyte, which was composed of gold (I) potassium cyanide (AuK (CN)_2_, 8 g L^–1^), potassium citrate (C_6_H_5_K_3_O_7_, 90 g L^–1^), citric acid (C_6_H_8_O_7_, 90 g L^–1^), and a brightener concentrate (10 mL L^–1^) that contained cobalt (II) carbonate as a grain refiner (0.05 g L^–1^). The beaker was placed onto a heating plate with an integrated magnetic mixer. The anode, a platinized titanium sheet, was fixed on the opposite of the holder facing the membrane. After wiring the electrodes to a potentiostat (Metrohm, Autolab PGSTAT101) and heating the solution to 35 °C while agitating the solution with a magnet stirrer at 250 rpm, the Au plug layer was plated at a DC current density −2 mA cm^–2^ and a pH 3.5. After the Au plug layer was plated, the holder stuck with the membrane was taken out and thoroughly rinsed with DI‐water and then dried by nitrogen. Afterward, Ni, Au, and Rh segments were electroplated by changing the electrolyte bath. Ni was electroplated at a pulse current density of −75 mA cm^–2^ and at 20 °C with a Ni electrolyte consisting of nickel (II) sulfate hexahydrate (NiSO_4_·6H_2_O, 300 g L^–1^) and boric acid as buffer agent (H_3_BO_3_, 45 g L^–1^). Rh was electroplated at a pulse current density of −100 mA cm^–2^ and at 20 °C from a commercial electrolyte (Technic, Inc. Co) containing rhodium (III) sulfate tetrahydrate (Rh_2_(SO_4_)_3_·4H_2_O). Nanowires with different lengths can be deposited by adjusting the plating time. After the electrodeposition, the membrane was rinsed three times with DI‐water and dried with nitrogen. To etch the sacrificial Au plug layer that served as the electrode at the bottom of the membrane, the membrane was placed in a beaker and submerged into a gold etchant GE‐8148 (Sigma‐Aldrich) for 5 min. This step dissolved only the Au plug layer but not the Au layer inside the membrane. After cleaning the membrane again, the nanowires were released by dissolving the AAO template with a 5 m NaOH solution. The AAO template was left for 2 h, at room temperature, inside the 5 m NaOH solution. This step completely dissolved the membrane and released the desired structures. The nanowires were rinsed three times with DI water and ethanol and transferred to 2 mL Eppendorf tubes. Note that, while Ni and Rh are not optimal in terms of biocompatibility and biodegradability, Ni and Rh were chosen due to their optimal fabrication and performance, which are satisfactory to demonstrate the motion capabilities of hinged nanoswimmers.

##### LbL Self‐Assembly Deposition

The nanowires were submerged in a 1 × 10^−3^ m aqueous MESA solution and agitated using a vortexer at 600 rpm for 30 min to form a negatively charged monolayer of MESA on the nanowires surface. After cleaning, the nanowires were suspended in a solution containing 1 mg mL^–1^ chitosan (CHI) and 0.5 m sodium chloride, and agitated at 600 rpm for 30 min. A positively charged polysaccharide CHI was bound to the MESA monolayer via electrostatic self‐assembly. Using the same method, the nanowires were immersed in a solution containing 1 mg mL^–1^ alginate (ALG) and 0.5 m NaCl, and agitated at 600 rpm for 30 min. A monolayer of negatively charged ALG was grown onto the nanowires surface by electrostatic self‐assembly. Following this procedure, bilayers of CHI and ALG can be alternately deposited on top of the nanowires until the desired thickness of polyelectrolyte layer was achieved. A thorough DI water rinse and a nitrogen blowing process were carried out after each polyelectrolyte layer deposition in order to prevent unwanted reactions generated by polyelectrolyte residues. For LBL deposition of PAH and PSS, the nanowires were immersed in 1 mg mL^–1^ PAH and 0.5 m NaCl solution, 1mg mL^–1^ sodium PSS and 0.5 m NaCl solution alternatively following the same process as described above for the formation of CHI and ALG bilayers. A Beckman Coulter – Delsa Nano C system was used to determine the *ζ*‐potential in each LbL deposition step. To simplify the *ζ*‐potential characterization, Ni–Au–Ni nanowires were used. The LbL process with PSS‐PAH‐based nanoswimmers was also monitored using fluorescein isothiocyanate (FITC)‐tagged PAH using Ni‐hinge‐Ni nanostructures. As shown in Figure S4 (Supporting Information), the fluorescent intensity of FITC molecules increases with increasing the number of bilayers. Figure S5 (Supporting Information) shows fluorescent images of nanoswimmers with different number of PAH/PSS bilayers captured by confocal laser scanning microscopy (Carl Zeiss). Note that the higher the number of deposited bilayers, the higher the stiffness of the coating, and subsequently the stiffness of the hinge. In this work, all the manipulation experiments were realized using 4‐bilayer hinges, except for those focused on evaluating the effect of the Ni head length, where only two bilayers were employed. No significant differences between stiffness were expected or noticed between 2 and 4 bilayers.^[^ ^26^ ^]^

##### Fabrication of the Flexible Hinge

After LbL self‐assembly process, the nanowires were immersed into gold etchant GE‐8148 (Sigma‐Aldrich) for 5 min to dissolve the Au sacrificial layer located between Ni head and Rh tail segments. All these etched nanoswimmers were rinsed by DI water for several times.

##### Characterization of the Hinged Nanoswimmers

Scanning electron microscopy (Zeiss ULTRA 55, Zeiss, Oberkochen, Germany) and transmission electron microscopy (FEI F30, FEI Co., Hillsboro, OR) images were taken to characterize the morphology of the hinged nanoswimmers.

##### Magnetic Manipulation Experiment

The swimming experiments were conducted with a Magnebotix Nanomag setup, which consisted of eight stationary electromagnetic coils, each wrapped around a soft magnetic core. This setup was able to produce a rotating magnetic field up to 50 mT and a magnetic frequency up to 100 Hz. The swimmers were immersed in 65% water–glycerol solution and placed in the center of eight electromagnetic coils to ensure a uniform magnetic field. 65% water–glycerol solution was chosen as it is one of the most widely used blood analog solutions, whose density (1160 kg m^−3^) and viscosity (5.3 mPa s) ^[^ ^27^ ^]^ are close to the values for blood (density: 1060 kg m^−3^, viscosity: 2.9–4.37 mPa s).^[^ ^28^ ^]^ By dynamically varying the magnetic field strength and frequency, the locomotion of nanoswimmers can be followed and captured by an inverted microscope (Olympus IX 81, Olympus Optical Co. Ltd., Japan).

##### Drug Loading Experiments

To use the hinged nanoswimmer as therapeutic cargo nanotransporters, a fluorescent model drug Rhodamine B was loaded within the polymeric hinge. To simplify the experiments and minimize possible changes of contrast between the two metallic segments under fluorescence microscope observation, nanoswimmers consisting of a Ni head, a polymeric hinge, and a short Ni tail were used. The nanoswimmers were dispersed in a RhB solution (1 mg mL^–1^) and the resulting dispersion was shaken for 12 h. Afterward, the nanoswimmers were thoroughly rinsed with DI water for several times to ensure no residual RhB molecules were stuck to the surface of the swimmers. The brightfield and fluorescence images of RhB‐loaded nanoswimmers were taken under the fluorescence microscope (Olympus IX‐81) by using a green filter to extract the fluorescent signal from RhB.

##### In Vitro Cell Experiments

The mouse 3T3 fibroblast cells were cultivated in 24‐well cell culture plates by using standard methods. The volume of cell culture medium in each well was 0.1 mL. Under physiological conditions, the cell medium (DMEM‐Glutamax) contains up to 10% fetal bovine serum. The mouse 3T3 fibroblasts cells were seeded and incubated at 37 °C for 4 h before any treatment. Afterward, a small phosphate buffered saline (PBS) solution containing nanoswimmers were delivered to the cell culture, which was placed under the fluorescence microscope. Images were taken by a fluorescent inverted optical microscope (Olympus IX‐81).

##### Statistical Analysis

Unless otherwise noted, at least five measurements (*n* = 5) were conducted for each set of nanoswimmers. The measured data were represented in form of average ± standard deviation.

## Conflict of Interest

The authors declare no conflict of interest.

## Supporting information

Supporting Information

Supplemental Video 1

Supplemental Video 2

Supplemental Video 3

Supplemental Video 4

Supplemental Video 5

Supplemental Video 6

Supplemental Video 7

Supplemental Video 8

Supplemental Video 9

Supplemental Video 10

## Data Availability

Research data are not shared.
