# Supplementary material for: Helical Klinotactic Locomotion of Two‐Link Nanoswimmers with Dual‐Function Drug‐Loaded Soft Polysaccharide Hinges
Source: Adv Sci (Weinh). 2021 Feb 15;8(8):2004458. doi: 10.1002/advs.202004458 (PMC8061375; doi:10.1002/advs.202004458)
Supplement: Supplementary file 1 — Supporting Information [file ADVS-8-2004458-s003.pdf]

WILEY-VCH

© 2021 Wiley-VCH GmbH

ADVANCED SCIENCE
Open Access

Supporting Information

for *Adv. Sci.,* DOI: 10.1002/advs.202004458

Helical Klinotactic Locomotion of Two-Link Nanoswimmers with Dual-Function Drug-Loaded Soft Polysaccharide Hinges

Jiaen Wu, Bumjin Jang, Yuval Harduf, Zvi Chapnik, Ö. Bartu Avci, Xiangzhong Chen, Josep Puigmartí-Luis, Olgac Ergeneman, Bradley J. Nelson, Yizhar Or,* and Salvador Pané**

Supporting information

Helical Klinotactic Locomotion of Two-Link Nanoswimmers with Dual-Function Drug-Loaded Soft Polysaccharide Hinges

Jiaen Wu, Bumjin Jang, Yuval Harduf, Zvi Chapnik, Ö. Bartu Avci, Xiangzhong Chen, Josep Puigmartí-Luis, Olgac Ergeneman, Bradley J. Nelson, Yizhar Or,* Salvador Pané**[1]

J. Wu, Dr. B. Jang, Ö. B. Avci, Dr. X. Chen, Dr. O. Ergeneman, Prof. B. J. Nelson, Prof. S. Pané
Multi-Scale Robotics Lab
Institute of Robotics and Intelligent Systems, ETH Zurich
Tannenstrasse3, CH-8092, Zurich, Switzerland
E-mail: vidalp@ethz.ch, silverjbj@gmail.com

Y. Harduf, Z. Chapnik, Prof. Y. Or
Faculty of Mechanical Engineering
Technion – Israel Institute of Technology, Haifa, 32000, Israel
E-mail: izi@me.technion.ac.il

Prof. J. Puigmarti-Luis
Departament de Ciència dels Materials i Química Física, Institut de Química Teòrica i Computacional, 08028 Barcelona, Spain.
ICREA, Pg. Lluís Companys 23, 08010 Barcelona, Spain.

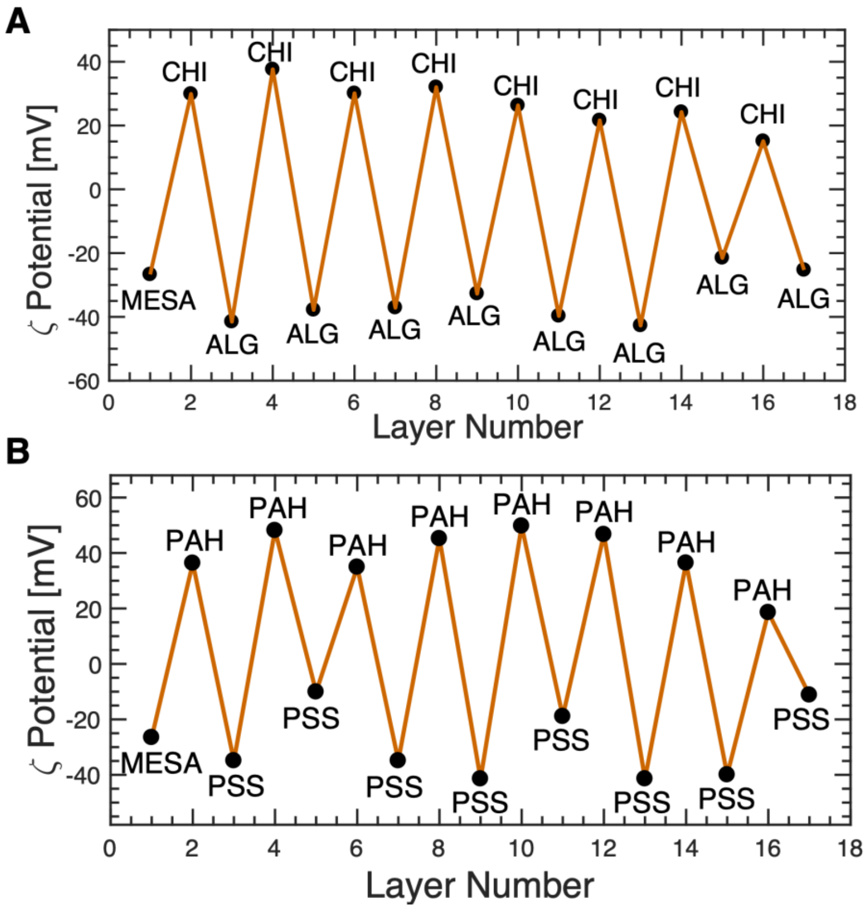

Figure S1. (A) ζ -potential for CHI and ALG deposition on nanowires. (B) ζ -potential for PAH and PSS deposition on nanowires.

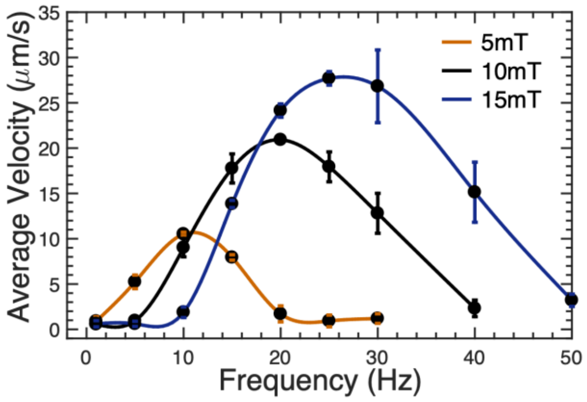

Figure S2. The average speed of hinged nanoswimmers with Ni head ~2 μm, PSS/PAH hinge ~2 μm, and Rh tail ~4 μm as a function of rotating magnetic frequency at field strength 5 mT, 10 mT, and 15 mT. The bar plot represents average ± SD (n = 5).

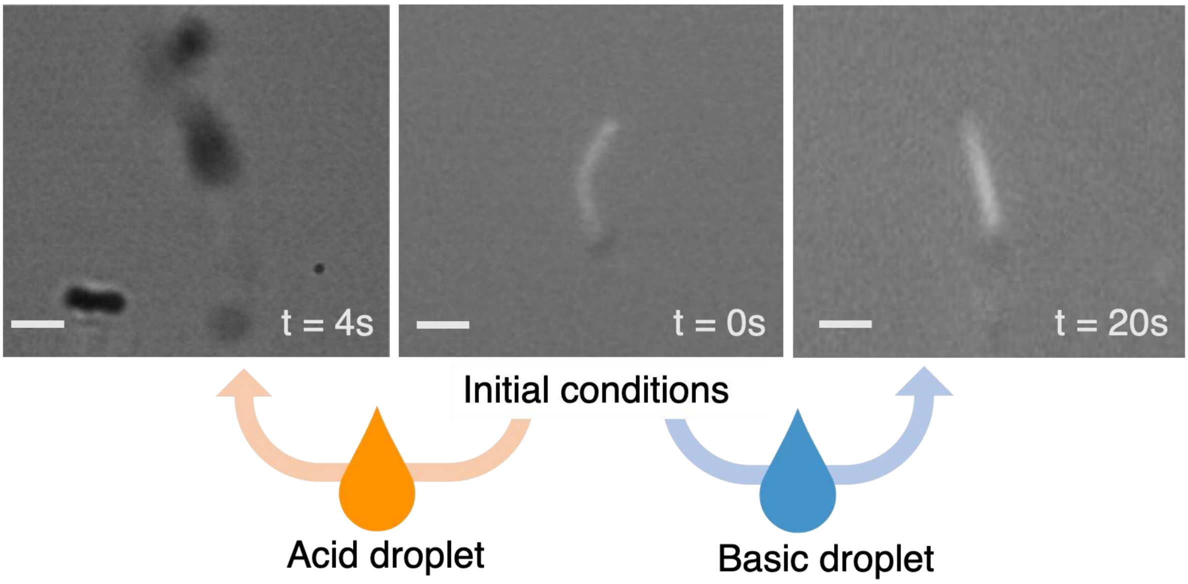

Figure S3. pH responsive drug release experiment with PAH/PSS hinge.

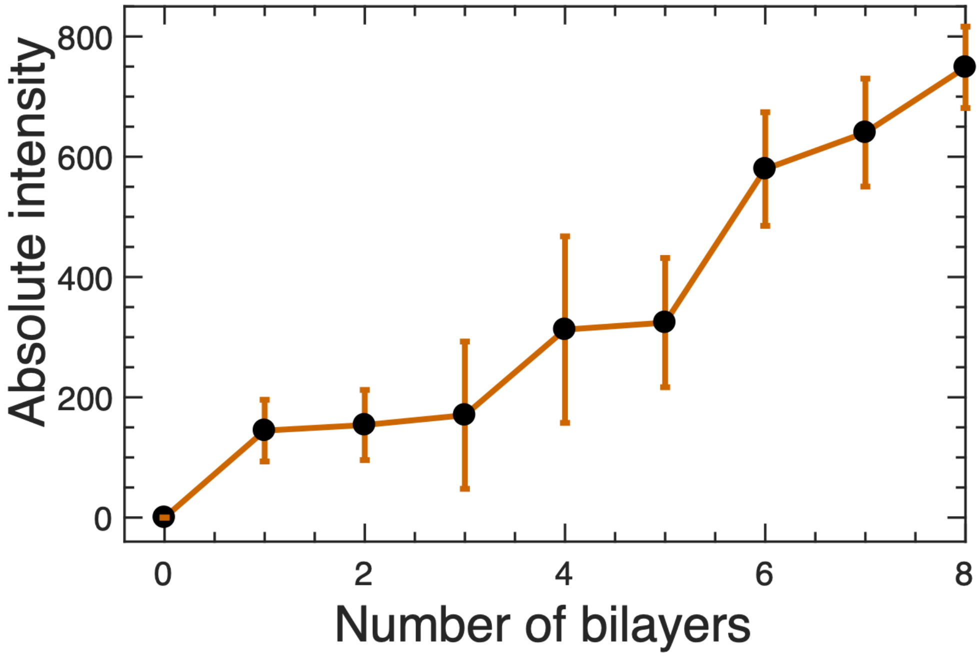

Figure S4. The absolute fluorescent intensity of FITC tagged PAH as a function of the number of PAH/PSS bilayers. The bar plot represents average ± SD (n = 5).

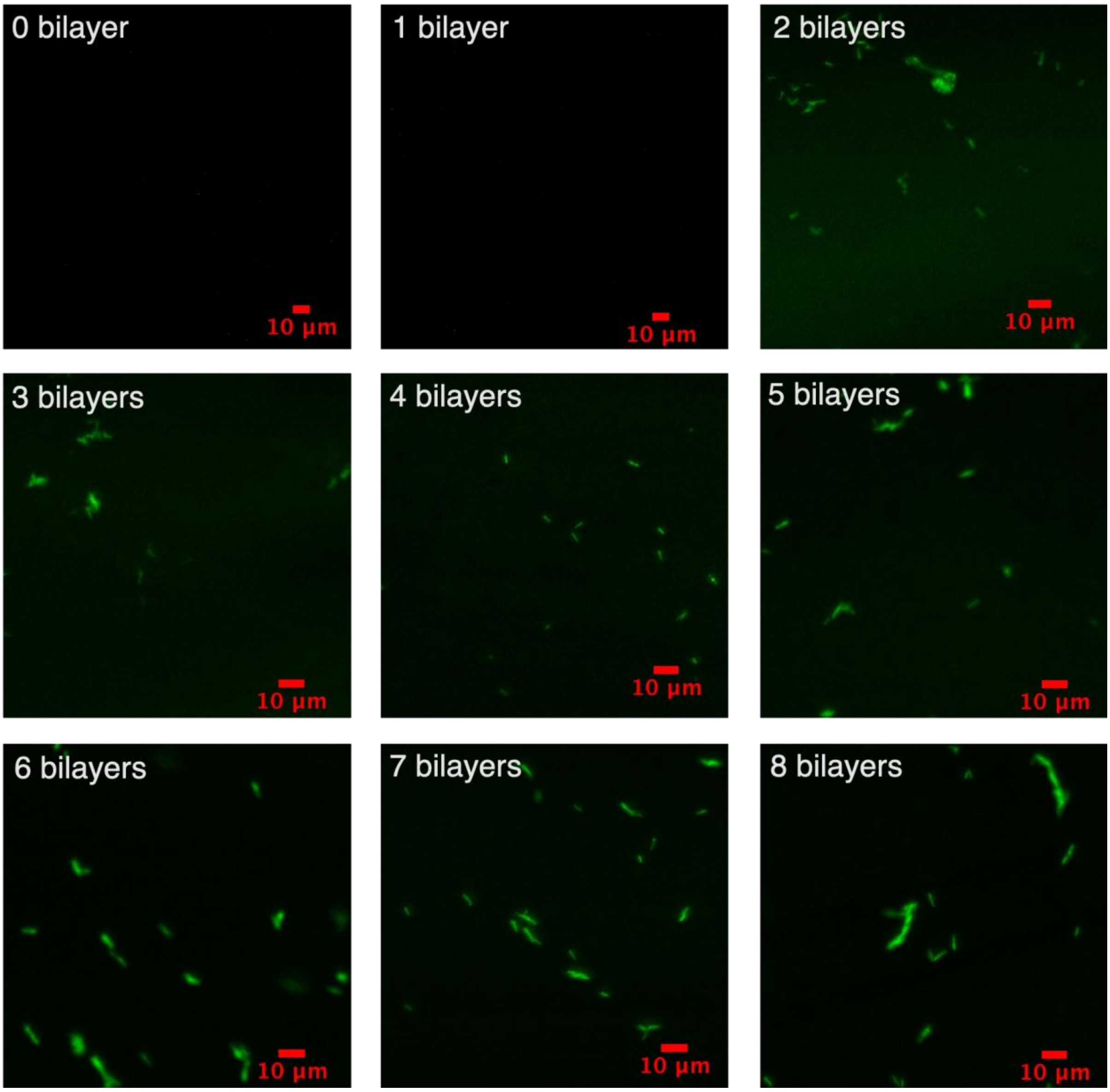

Figure S5. CLSM fluorescent images of different number of PAH/PSS bilayers hinges incubated with FITC-PAH.

Modelling

The nanoswimmer body coordinates vector is defined as $\mathbf{q}_b = (x \quad y \quad z \quad \phi \quad \theta \quad \psi)^T$, the shape coordinates vector is defined by $\mathbf{q}_s = (\delta)$. The overall coordinates vector

$\mathbf{q} = \begin{pmatrix} \mathbf{q}_b & \mathbf{q}_s \end{pmatrix}^T$. The orientation matrix of the head link is obtained as the product of three extrinsic rotation matrices according to the ZXZ convention as $\mathbf{R}_1 = \mathbf{R}_\phi \mathbf{R}_\theta \mathbf{R}_\psi$, where

$$\mathbf{R}_\phi = \begin{pmatrix} \cos(\phi) & -\sin(\phi) & 0 \\ \sin(\phi) & \cos(\phi) & 0 \\ 0 & 0 & 1 \end{pmatrix}, \mathbf{R}_\theta = \begin{pmatrix} 1 & 0 & 0 \\ 0 & \cos(\theta) & -\sin(\theta) \\ 0 & \sin(\theta) & \cos(\theta) \end{pmatrix}, \mathbf{R}_\psi = \begin{pmatrix} \cos(\psi) & -\sin(\psi) & 0 \\ \sin(\psi) & \cos(\psi) & 0 \\ 0 & 0 & 1 \end{pmatrix}$$

The rotation matrix representing the orientation of the non-magnetic tail link is obtained by an additional rotation angle δ around the $\hat{\boldsymbol{z}}_1$ axis, hence:

$$\mathbf{R}_2 = \mathbf{R}_\phi \mathbf{R}_\theta \mathbf{R}_{\psi+\delta}, \text{ where } \mathbf{R}_{3+\delta} = \begin{pmatrix} \cos(\psi+\delta) & -\sin(\psi+\delta) & 0 \\ \sin(\psi+\delta) & \cos(\psi+\delta) & 0 \\ 0 & 0 & 1 \end{pmatrix}.$$

The longitudinal axes of each link are denoted by $\hat{\mathbf{t}}_1, \hat{\mathbf{t}}_2$, which can be obtained by taking the first column of the transformation matrices $\mathbf{R}_1$ and $\mathbf{R}_2$.

The positions of each link's center are denoted as $\mathbf{r}_1 = \begin{pmatrix} x & y & z \end{pmatrix}^T, \mathbf{r}_2 = \mathbf{r}_1 - \frac{l_1}{2}\hat{\mathbf{t}}_1 - \frac{l_2}{2}\hat{\mathbf{t}}_2$. The position of the joint is denoted as $\mathbf{r}_j = \mathbf{r}_1 - \frac{l_1}{2}\hat{\mathbf{t}}_1$. The velocity vector of the ith point on the link can be defined as $\mathbf{V}_i = \begin{pmatrix} \mathbf{v}_i & \boldsymbol{\omega}_i \end{pmatrix}^T$, where $\mathbf{v}_i, \boldsymbol{\omega}_i$ are the linear and angular velocity vectors. The shape velocity vector is denoted as $\mathbf{u} = \left(\dot{\mathbf{q}}_s\right)$ and the body velocity vector is denoted as $\mathbf{V}_b = \begin{pmatrix} \mathbf{v}_b & \boldsymbol{\omega}_b \end{pmatrix}^T$.

Using rigid-body kinematic relations, the link's velocity can be expressed by the body and shape velocity as (cf. [S1]):

$$\mathbf{V}_i = \mathbf{T}_i \mathbf{V}_b + \mathbf{E}_i \mathbf{u}$$

where

$$\mathbf{T}_1 = \mathbf{I}_{6\times6}, \mathbf{E}_1 = \mathbf{0}_{6\times1}, \mathbf{T}_2 = \begin{pmatrix} \mathbf{I}_{3\times3} & -\left[(\mathbf{r}_2 - \mathbf{r}_1)\times\right] \\ \mathbf{0}_{3\times3} & \mathbf{I}_{3\times3} \end{pmatrix}, \mathbf{E}_2 = \begin{pmatrix} \hat{\mathbf{z}}_1 \times (\mathbf{r}_2 - \mathbf{r}_j) \\ \hat{\mathbf{z}}_1 \end{pmatrix}$$

$\hat{\mathbf{z}}_1$ is the 3rd column of $\mathbf{R}_1$. The notation $[(a)]\times$ represents cross-product in matrix form as:

$$\mathbf{a}\times\mathbf{b} = \left([(\mathbf{a})]\times\right)\cdot\mathbf{b},\ [(\mathbf{a})]\times = \begin{pmatrix} 0 & -a_3 & a_2 \\ a_3 & 0 & -a_1 \\ -a_2 & a_1 & 0 \end{pmatrix}$$

The relation between each link's angular velocities and the derivatives of the rotational body coordinates can be written as:

$$\boldsymbol{\omega}_b = \dot{\phi}\begin{pmatrix} 0 \\ 0 \\ 1 \end{pmatrix} + \dot{\theta}\begin{pmatrix} \cos(\phi) \\ \sin(\phi) \\ 0 \end{pmatrix} + \dot{\psi}\begin{pmatrix} \sin(\theta)\sin(\phi) \\ -\sin(\theta)\cos(\phi) \\ \cos(\theta) \end{pmatrix}$$

$$\boldsymbol{\omega}_b = \underbrace{\begin{pmatrix} 0 & \cos(\phi) & \sin(\theta)\sin(\phi) \\ 0 & \sin(\phi) & -\cos(\phi)\sin(\theta) \\ 1 & 0 & \cos(\theta) \end{pmatrix}}_{\mathcal{W}}\begin{pmatrix} \dot{\phi} \\ \dot{\theta} \\ \dot{\psi} \end{pmatrix}$$

Then the links' velocities can thus be written as:

$$\mathbf{V}_i = \mathbf{T}_i\underbrace{\begin{pmatrix} \mathbf{I}_{3\times3} & \mathbf{0}_{3\times3} \\ \mathbf{0}_{3\times3} & \mathcal{W} \end{pmatrix}}_{\mathcal{K}}\dot{\mathbf{q}}_b + \mathbf{E}_i\dot{\mathbf{q}}_s$$

$$\mathbf{V}_i = \mathbf{T}_i\mathcal{K}\dot{\mathbf{q}}_b + \mathbf{E}_i\dot{\mathbf{q}}_s$$

Forces and actuation

Hydrodynamics:

The swimmer's links are modeled as prolate spheroids. By using following drag coefficients: $c_t^i, c_n^i, c_{rt}^i, c_{rn}^i$, which are the linear and rotational coefficients along and normal to the longitudinal axis of the ith link, the forces acting upon each link can be expressed as follows:

$$\mathbf{f}^h_i = -c_t^i\left(\mathbf{v}_i\cdot\hat{\mathbf{t}}_i\right)\hat{\mathbf{t}}_i - c_n^i\left(\mathbf{v}_i - \left(\mathbf{v}_i\cdot\hat{\mathbf{t}}_i\right)\hat{\mathbf{t}}_i\right) = \left(\left(c_n^i - c_t^i\right)\hat{\mathbf{t}}_i\cdot\hat{\mathbf{t}}_i^T - c_n^i\mathbf{I}_{3\times3}\right)\mathbf{v}_i$$

$$\boldsymbol{\tau}^h_i = -c_{rt}^i\left(\boldsymbol{\omega}_i\cdot\hat{\mathbf{t}}_i\right)\hat{\mathbf{t}}_i - c_{rn}^i\left(\boldsymbol{\omega}_i - \left(\boldsymbol{\omega}_i\cdot\hat{\mathbf{t}}_i\right)\hat{\mathbf{t}}_i\right) = \left(\left(c_{rn}^i - c_{rt}^i\right)\hat{\mathbf{t}}_i\cdot\hat{\mathbf{t}}_i^T - c_{rn}^i\mathbf{I}_{3\times3}\right)\boldsymbol{\omega}_i$$

The hydrodynamic forces and torques acting on each link depend on velocities as $\mathbf{F}_i^h = \begin{pmatrix} \mathbf{f}_i^h \\ \boldsymbol{\tau}_i^h \end{pmatrix} = -\mathcal{R}_i \mathbf{V}_i$, where the resistance tensors $\mathcal{R}_i$ are defined as:

$$\mathcal{R}_i = \begin{pmatrix} \left(c_t^i - c_n^i\right)\hat{\mathbf{t}}_i \cdot \hat{\mathbf{t}}_i^T + c_n^i \mathbf{I}_{3\times3} & \mathbf{0}_{3\times3} \\ \mathbf{0}_{3\times3} & \left(c_{rt}^i - c_{rn}^i\right)\hat{\mathbf{t}}_i \cdot \hat{\mathbf{t}}_i^T + c_{rn}^i \mathbf{I}_{3\times3} \end{pmatrix}$$

The links are modelled as prolate spheroids with major radius $a_i=0.5l_i$ and minor radii b_i. We use known formulas for drag coefficients of translational [S2] and rotational [S3, S4] motion , as:

$$c_t^i = \frac{4\pi\mu a_i}{log\left(\frac{2a_i}{b_i} - 0.5\right)}, c_n^i = \frac{8\pi\mu a_i}{log\left(\frac{2a_i}{b_i} + 0.5\right)}, c_{rt}^i = \frac{2\mu V_i}{n_\perp^i}, c_{rn}^i = 2\mu V_i \left(\frac{a_i^2 + b_i^2}{a_i^2 n_\parallel^i + b_i^2 n_\perp^i}\right)$$

where $V_i = \frac{4\pi}{3} a_i {b_i}^2$ is the spheroid's volume, $e_i = \sqrt{1 - \frac{{b_i}^2}{{a_i}^2}}$ is its eccentricity, $n_\parallel^i = \frac{1-{e_i}^2}{2{e_i}^3} log\left(\frac{1+e_i}{1-e_i} - e_i\right), n_\perp^i = \frac{1-n_\parallel^i}{2}$, and μ is the fluid's viscosity.

Magnetism:

The applied rotating magnetic field is expressed as:

$$\mathbf{B} = B \cdot \begin{pmatrix} 0 & \sin(\omega t) & \cos(\omega t) \end{pmatrix}^T$$

The torque acting on the head link due to the rotating magnetic field is $\boldsymbol{\tau}^m = \boldsymbol{MV}(\hat{\boldsymbol{t}}_1 \times \mathbf{B})$, where $\boldsymbol{V} = \frac{\pi}{4} l_1 {a_1}^2$ is the link volume calculated as a cylinder, and $\boldsymbol{M}$ is the magnetic moment per unit volume.

Therefore, the vectors of magnetic forces and torques on each link are:

$$\mathbf{F}_1^m = \begin{pmatrix} \mathbf{0}_{3\times1} \\ \boldsymbol{\tau}^m \end{pmatrix}, \mathbf{F}_2^m = \mathbf{0}_{6\times1}$$

Elasticity

The spring applies a torque relative to the joint's angle:

$$\tau_k = -k\delta$$

Motion equations

The swimmer is always in force and torque balance. Since the motion is governed by low Reynolds number hydrodynamics, there is no inertia. $\mathbf{T}_i, \mathbf{E}_i$ are used to express the balance of net external forces and torques acting on the swimmer's body as (cf. [S1]):

$$\sum_{i=1}^{2} \mathbf{T}_i^T \left(\mathbf{F}_i^h + \mathbf{F}_i^m \right) = \sum_{i=1}^{2} \mathbf{T}_i^T \left(-\mathcal{R}_i \mathbf{V}_i + \mathbf{F}_i^m \right) = \sum_{i=1}^{2} \mathbf{T}_i^T \left(-\mathcal{R}_i \left(\mathbf{T}_i \mathcal{K} \dot{\mathbf{q}}_b + \mathbf{E}_i \dot{\mathbf{q}}_s \right) + \mathbf{F}_i^m \right) =$$
$$\sum_{i=1}^{2} \left(-\mathbf{T}_i^T \mathcal{R}_i \mathbf{T}_i \mathcal{K} \dot{\mathbf{q}}_b - \mathbf{T}_i^T \mathcal{R}_i \mathbf{E}_i \dot{\mathbf{q}}_s \right) + \sum_{i=1}^{2} \mathbf{T}_i^T \mathbf{F}_i^m = 0$$
$$\rightarrow \underbrace{\sum_{i=1}^{2} \mathbf{T}_i^T \mathcal{R}_i \mathbf{T}_i}_{\mathcal{R}_{bb}} \mathcal{K} \dot{\mathbf{q}}_b + \underbrace{\sum_{i=1}^{2} \mathbf{T}_i^T \mathcal{R}_i \mathbf{E}_i}_{\mathcal{R}_{bu}} \dot{\mathbf{q}}_s = \sum_{i=1}^{2} \mathbf{T}_i^T \mathbf{F}_i^m$$

In addition, torque balance at the joint can be written as:

$$\sum_{i=1}^{2} \mathbf{E}_i^T \left(\mathbf{F}_i^h + \mathbf{F}_i^m \right) + \tau_k = \sum_{i=1}^{2} \mathbf{E}_i^T \left(-\mathcal{R}_i \mathbf{V}_i + \mathbf{F}_i^m \right) + \tau_k = \sum_{i=1}^{2} \mathbf{E}_i^T \left(-\mathcal{R}_i \left(\mathbf{T}_i \mathcal{K} \dot{\mathbf{q}}_b + \mathbf{E}_i \dot{\mathbf{q}}_s \right) + \mathbf{F}_i^m \right) + \tau_k =$$
$$\sum_{i=1}^{2} \left(\left(-\mathbf{E}_i^T \mathcal{R}_i \mathbf{T}_i \mathcal{K} \dot{\mathbf{q}}_b - \mathbf{E}_i^T \mathcal{R}_i \mathbf{E}_i \dot{\mathbf{q}}_s \right) + \mathbf{E}_i^T \mathbf{F}_i^m \right) + \tau_k = 0$$
$$\rightarrow \underbrace{\sum_{i=1}^{2} \mathbf{E}_i^T \mathcal{R}_i \mathbf{T}_i}_{\mathcal{R}_{bu}^T} \mathcal{K} \dot{\mathbf{q}}_b + \underbrace{\sum_{i=1}^{2} \mathbf{E}_i^T \mathcal{R}_i \mathbf{E}_i}_{\mathcal{R}_{uu}} \dot{\mathbf{q}}_s = \sum_{i=1}^{2} \mathbf{E}_i^T \mathbf{F}_i^m + \tau_k$$

The two equations above can be written in matrix form as:

$$\underbrace{\begin{pmatrix} \mathcal{R}_{bb} \mathcal{K} & \mathcal{R}_{bu} \\ \mathcal{R}_{bu}^T \mathcal{K} & \mathcal{R}_{uu} \end{pmatrix}}_{\mathbf{A}} \begin{pmatrix} \dot{\mathbf{q}}_b \\ \dot{\mathbf{q}}_s \end{pmatrix} = \underbrace{\begin{pmatrix} \sum_{i=1}^{2} \mathbf{T}_i^T \mathbf{F}_i^m \\ \sum_{i=1}^{2} \mathbf{E}_i^T \mathbf{F}_i^m + \tau_k \end{pmatrix}}_{\mathbf{b}}, \text{ or } \mathbf{A}(\mathbf{q})\dot{\mathbf{q}} = \mathbf{b}(\mathbf{q}, t)$$

This gives a system of nonlinear ordinary differential equations, which are integrated numerically in order to obtain the solution $\mathbf{q}(t)$, from which the swimmer's motion can be extracted.

Physical values for the model parameters were chosen as follows:

Magnetic field strength study:

(i) B = 5mT, M = 90000 A/m, l_1 = 3.1 μm, l_2 = 4.5 μm, μ = 0.0124 N•s/m^2, k = 3.75×10^{-17} N•m.

(ii) B = 10mT, M = 90000 A/m, l_1 = 3.1 μm, l_2 = 4.5 μm, μ = 0.0124 N•s/m^2, k = 3.75×10^{-17} N•m.

(iii) B = 15mT, M = 90000 A/m, l_1 = 3.1 μm, l_2 = 4.5 μm, μ = 0.0124 N•s/m^2, k = 3.75×10^{-17} N•m.

Hinge length study:

(i) B = 5mT, M = 90000 A/m, l_1 = 3.1 μm, l_2 = 4.7 μm, μ = 0.0124 N•s/m^2, k = 3.75×10^{-17} N•m.

(ii) B = 5mT, M = 90000 A/m, l_1 = 3.6 μm, l_2 = 5.2 μm, μ = 0.0124 N•s/m^2, k = 1.875×10^{-17} N•m.

(iii) B = 5mT, M = 90000 A/m, l_1 = 4.6 μm, l_2 = 5.6 μm, μ = 0.0124 N•s/m^2, k = 1.25×10^{-17} N•m.

Ni head length study:

(i) B = 5mT, 10mT, M = 90000 A/m, l_1 = 4.4 μm, l_2 = 6.7 μm, μ = 0.0124 N•s/m^2, k = 3.25×10^{-17} N•m.

(ii) B = 5mT, 10mT, M = 90000 A/m, l_1 = 7.2 μm, l_2 = 6.5 μm, μ = 0.0124 N•s/m^2, k = 3.25×10^{-17} N•m.

where M is the magnetic moment, μ is 65% glycerol solution viscosity.

References:

[S1] Wiezel, O., & Or, Y. (2016). Optimization and small-amplitude analysis of Purcell's three-link microswimmer model. Proceedings of the Royal Society A: Mathematical, Physical and Engineering Sciences, 472(2192), 20160425.

[S2] Happel, J., & Brenner, H. (1983). Low Reynolds number hydrodynamics: with special applications to particulate media (Vol. 1). Springer Science & Business Media.

[S3] Morozov, K. I., & Leshansky, A. M. (2014). The chiral magnetic nanomotors. Nanoscale, 6(3), 1580-1588.

[S4] G. B. Jeffrey (1922). The motion of ellipsoidal particles immersed in a fluid. Proc. R. Soc. London A, 102, 161–179.
